# Effect of hemodialysis fluid cooling uremic pruritus in hemodialysis patients

**Published:** 2012-07-01

**Authors:** Mohammad-Reza Tamadon

**Affiliations:** Department of Internal Medicine, Semnan University of Medical Sciences, Semnan, Iran

**Keywords:** Pruritus, Hemodialysis, Uremia

**Implication for health policy/practice/research/medical education:**

Pruritus can be one of the important factors of exacerbation of discomfort in patients with chronic renal failure. In an unpublished clinical trial which was performed in the Semnan University of Medical Sciences, the effect of reduction of dialysate temperature on controlling the pruritus was assessed. The results suggest a positive effect. The author recommends further studies to consider reducing dialysate temperature on control of pruritus.

Pruritus is a common problem in patients with chronic renal failure 25 to 35% before dialysis patients and 60 to 80% of dialysis patients may complain of pruritus (1). Despite advances in dialysis techniques and the treatment of uremic pruritus, it is remains an uncontrolled clinical problem in patients with chronic kidney disease (2). Results of a study have shown a 48% prevalence of pruritus (3). Also, in another study conducted by Yaghobi and colleagues in Iran, incidence of pruritus was 58% in hemodialysis patients (4). Pathophysiology of pruritus in patients with chronic renal failure is unknown and various hypotheses have been proposed. One of the most accepted hypothesis is the histamine hypothesis. Regardless of the mechanism of pruritus, symptoms of pruritus in dialysis patients has close relation with histamine release from mast cells of the skin. Studies have shown that in uremic patients, the number of basophiles and coetaneous mast cells was increased (5). Various studies have shown that uremic pruritus is a systemic disease and is not only limited to skin and in a study has been shown that ultraviolet relieve uremic pruritus in patients and have regulatory effect on uremic action so, the immune hypothesis propounded for pruritus (6). Another strong hypothesis in uremic pruritus, is opioid theory. One of the side effects of μ receptors stimulator drugs, especially when administrate as systemic, is pruritus. In 1985, the first successful treatment of uremic pruritus was reported following intravenous administration of naloxone (7).

The use of opioid antagonists in treatment of uremic pruritus have been based on this matter that endogenous opioid peptides accumulate in uremic patients and its concentration increases in plasma. In a controlled study the effects of naltrexone on pruritus in hemodialysis patients was studied and showed a positive effect in these patients, although another study showed controversy result (8). In a study, hydroxyzine and Chlorpheniramine was used for control of pruritus which has been effective (9). As mentioned above, the mechanism of pruritus in dialysis patients is very complex and many factors affect its alleviation and intensification effective.

There is an important matter which is in addition to other problems; pruritus can be one of the important factors of exacerbation of discomfort in patients with chronic renal failure. In an unpublished clinical trial that was performed in the Semnan University of Medical Sciences, the effect of reduction of dialysate temperature on controlling the pruritus was assessed. The results suggest a positive effect (10). Some systemic effects of reducing dialysate temperature and ambient temperature on controlling of pruritus have been examined (10,11). The author recommends further studies to consider reducing dialysate temperature on control of pruritus.

Conclusion

Pruritus can be one of the important factors of exacerbation of discomfort in patients with chronic renal failure. In an unpublished clinical trial which was performed in the Semnan University of Medical Sciences, the effect of reduction of dialysate temperature on controlling the pruritus was assessed. The results suggest a positive effect. The author recommends further studies to consider reducing dialysate temperature on control of pruritus.

## Author’s contribution

MRT was the single author of the paper.

## Conflict of interests

The author declared no competing interests.

## Ethical considerations

Ethical issues (including plagiarism, data fabrication, double publication) have been completely observed by the author.

## Funding/Support

None.
